# Ping-Pong Positioning: Alternating Protein Interactions at Actin Filament Barbed Ends Helps Establish Polarity in Mammalian Oocytes

**DOI:** 10.1371/journal.pbio.1001796

**Published:** 2014-02-25

**Authors:** Mary Hoff

**Affiliations:** Freelance Science Writer, Stillwater, Minnesota, United States of America

[Fig pbio-1001796-g001]For mammalian egg cells to form successfully, the precursor cell (the oocyte) must divide asymmetrically, forming a large egg that contains the storage material required for embryo development, and a small polar body that receives surplus chromosomes. How does the oocyte manage this asymmetrical division? Key to the answer is a meshwork of actin filaments that moves the chromosome-segregating spindle, initially formed at the center of the oocyte, toward the cell cortex.

**Figure pbio-1001796-g001:**
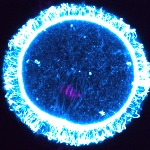
Mouse oocyte, showing the spindle undergoing translocation toward the cortex. Actin network - cyan, chromosomes - magenta. *Image credit: Binyam Mogessie and Melina Schuh.*

The meshwork-building process involves three actin-binding proteins named profilin, Formin 2, and Spire (also known to display genetic interactions in Drosophila), which cooperate in vivo. But exactly how do these three proteins work together to accomplish this important task? A new in vitro study by Marie-France Carlier, Pierre Montaville, and colleagues has revealed a fascinating ping-pong like interaction between Formin 2 and Spire acting on profilin-bound actin.

It was known that the C-terminus of the FH2 domain of Formin 2 associates with the N-terminal (Nt) KIND domain of Spire. The present team previously found that when Nt-Spire alone (composed of the KIND domain and 4 WH2 domains) associates with actin barbed ends via its WH2 domains, it blocks their growth from profilin-actin. On the other hand, Formin 2 (like other formins), is expected to promote processive filament barbed end assembly from profilin-actin. Thus Spire and Formin 2 individually have antagonistic effects on actin assembly. How then can synergistic actin assembly arise from the two proteins together?

The authors here use a combination of bulk solution and single filament (microfluidics-assisted TIRF microscopy) polymerization assays to reconstitute synergistic actin assembly in vitro and provide mechanistic insight. They show that the FH1-FH2 domain of Formin 2 by itself poorly nucleates filaments from profilin-actin and Nt-Spire alone inhibits assembly, but together they display enhanced assembly. Barbed end growth assays establish that the two proteins interact together at filament barbed ends to promote this synergy, and that their direct mutual interaction is mediated by association of the KIND domain of Nt-Spire with the FH2 C-terminal region of Formin 2. However by itself, the KIND domain inhibits filament assembly. Thus the WH2 domains of Spire are involved in the synergistic behavior.

The team also demonstrates that *in vitro*, at steady state conditions mimicking the *in vivo* context, the amount of assembled actin is determined by the relative amounts of Spire and Formin 2.

The exact molecular mechanism responsible for the puzzling synergistic effect of Spire and Formin 2 is established by single filament assays. The team shows that while individual filaments grow slowly from profilin-actin in the absence of Spire or Formin 2, they switch to arrested growth when the barbed ends are capped by Spire. Formin 2 binds unusually slowly to barbed ends, but when it does it promotes very fast processive assembly. Remarkably, Formin 2 binds 30-fold faster to Spire-capped ends than to free barbed ends, where it is recruited by Spire's KIND domain. Spire actually saddles Formin 2 at the barbed ends, promoting fast processive assembly, which accounts for the synergistic assembly. Spire then rapidly dissociates from the barbed end. Conversely, association of Spire to a Formin 2-bound barbed end arrests fast growth. When Spire and Formin 2 are present together in solution, the filaments display alternating phases of fast and arrested growth, corresponding to alternating Formin 2-bound and Spire-bound states, each protein kicking the other off via a transient state in which both are bound to the filament end. The exact kick-off mechanism remains unknown but may involve ATP hydrolysis on actin. Indeed, filament depolymerization assays show that Spire and Formin 2 bind together cooperatively at depolymerizing ADP-bound barbed ends and block disassembly.

Finally, the team looked at the system *in vivo* by injecting oocytes with Nt-Spire, the KIND domain of Spire, or the FH1-FH2 domains of Formin 2. They found that Nt-Spire alone or FH1-FH2 alone caused an increase in actin filament growth, while KIND alone had the opposite effect and FH2 depressed filament assembly. These data are consistent with and validate the relevance of *in vitro* studies.

But how does this facilitate asymmetric division? The answer to that has to do with the recently observed presence of Formin 2 and Spire on Rab11a positive vesicles in oocytes, and the associated myosinVb-promoted vesicle movement toward the oocyte cortex. The authors tentatively propose that Rab11a-vesicles constantly initiate new filaments via co-association of Spire and Formin 2 to barbed ends. At the same time, the other end of filaments disassembles, creating a pool of profilin-actin fueling filament assembly. The plasticity and myosinVb-enhanced dynamics of this treadmilling network facilitate the slow displacement of the spindle toward the cortex, breaking the symmetry that ultimately leads to egg development.

**Montaville P, Jégou A, Pernier J, Compper C, Guichard B, et al. (2014) Spire and Formin 2 Synergize and Antagonize in Regulating Actin Assembly in Meiosis by a Ping-Pong Mechanism.** doi:10.1371/journal.pbio.1001795

